# The efficacy and safety of anti-interleukin-6 receptor monoclonal blockade in a renal transplant patient with Castleman disease: early post-transplant outcome

**DOI:** 10.1186/s12882-018-1065-4

**Published:** 2018-10-11

**Authors:** Masatoshi Matsunami, Yoshifumi Ubara, Keiichi Sumida, Yoichi Oshima, Masahiko Oguro, Kazuya Kinoshita, Kiho Tanaka, Yuki Nakamura, Keiichi Kinowaki, Kenichi Ohashi, Takeshi Fujii, Takuro Igawa, Yasuharu Sato, Yasuo Ishii

**Affiliations:** 1 Department of Surgery, Nephrology Center, Toranomon Hospital, 2-2-2 Toranomon, Minato-ku, Tokyo, 105-8470 Japan; 2 Nephrology Center, Toranomon Hospital, Tokyo, Japan; 3 Department of Pathology, Toranomon Hospital, Tokyo, Japan; 4 Okinaka Memorial Institute for Medical Research, Tokyo, Japan; 5 Department of Pathology, Yokohama City University Graduate School of Medicine, Yokohama, Japan; 6 Department of Pathology, Okayama University Graduate School of Medicine, Dentistry, and Pharmaceutical Sciences, Okayama, Japan

**Keywords:** Castleman disease, Kidney transplantation, Tocilizumab, IL-6, IgA nephropathy

## Abstract

**Background:**

Multicentric Castleman disease (MCD) is an uncommon lymphoproliferative disease characterized by systemic inflammatory reactions associated with the dysregulated production of interleukin-6 (IL-6). In patients with MCD, renal involvement is uncommon, with only one report published regarding kidney transplantation (KTx) to treat end-stage renal disease (ESRD) secondary to MCD. Recent clinical observations have shown that IL-6 production is implicated in allograft rejection, while IL-6 receptor blockade (with tocilizumab [TCZ]) reduces alloantibody generation and thereby improves graft survival; however, the efficacy and safety of TCZ in MCD patients undergoing KTx is still unknown.

**Case presentation:**

Herein, we describe the case of a 50-year-old man with MCD who received living-donor KTx for ESRD. Post-operative immunosuppression consisted of a triple-drug regimen (tacrolimus, mycophenolate mofetil and methylprednisolone) with TCZ that was administered intravenously every 2 weeks. At 17 months post-transplantation, the patient remains asymptomatic, and the allograft pathology has shown no evidence of rejection and no development of de novo donor-specific antibody (DSA).

**Conclusions:**

To our knowledge, this is the second reported case of an MCD patient with ESRD who underwent successful KTx. TCZ safely supported the patient during the perioperative period, and this drug may be useful for blocking the generation of donor-specific antibodies and reducing the risk of rejection episodes. KTx in combination with TCZ is thus considered a viable treatment option for ESRD due to MCD.

## Background

Castleman disease (CD) is an uncommon heterogeneous group of lymphoproliferative disorders with three histological types (hyaline vascular type, plasma cell type, and mixed type). CD can occur in a localized (unicentric CD: UCD) or widespread (multicentric CD: MCD) form. The aetiology of CD is not well understood; however, excessive production of interleukin 6 (IL-6) by lymph node hyperplasia has been implicated in its pathogenesis [1–3]. The clinical features include generalized lymphadenopathy, organomegaly, and systemic manifestations, such as fatigue, a low-grade fever, and weight loss. Abnormal laboratory findings include anaemia, hypoalbuminaemia, hypocholesterolaemia, hypergammaglobulinaemia, and increased levels of C-reactive protein (CRP) [1–3]. Renal involvement in MCD seems to be uncommon and has been described in only a few case series [4, 5]. To date, there has been only one report of kidney transplantation (KTx) for the treatment of end-stage renal disease (ESRD) secondary to MCD [6].

IL-6 is a key cytokine that impacts the development and maturation of T cells, B cells, and antibody producing plasma cells. Excessive IL-6 production has been linked to several human diseases characterized by unregulated antibody production and autoimmunity, such as CD [1, 2]. Tocilizumab (TCZ; Actemra®, Roche/Genentech, San Francisco, CA, USA) is a first-in-class humanized monoclonal blockade targeting the IL-6 receptor (IL-6R). TCZ binds to both soluble and membrane-bound forms of the IL-6R and is approved for the treatment of CD [1, 2].

A recent study of TCZ for patients undergoing KTx showed a significant reduction in donor-specific antibody (DSA) production and improvement in the transplantation graft survival [7, 8]. IL-6R interactions have been shown to be critical for alloantibody generation in an animal model of alloimmunity. IL-6 modulates T cell immunity and is a powerful stimulant of pathogenic IgG production, so the blockade of these interactions with an anti–IL-6R monoclonal blockade results in significant reductions in alloantibodies [7, 8]. However, the efficacy and safety of TCZ in MCD patients undergoing KTx is still unknown. To answer this question, we herein report the second known case of an MCD patient with ESRD who successfully underwent living-donor KTx using TCZ.

## Case presentation

A 33-year-old man visited an outpatient clinic with haematuria and proteinuria. One year later, the patient was diagnosed with IgA nephropathy by a renal biopsy and was treated with diet and medication. At 36 years of age, his began to experience fatigue and weight loss. He was had generalized lymphadenopathy, hepatosplenomegaly, and elevated CRP levels, and his IL-6 level had gradually increased. Chest CT revealed slight enlargement of the mediastinal lymph nodes, centrilobular nodules, thickening of the bronchovascular bundles, and ground-glass opacities. These clinical findings were consistent with MCD. Hepatitis B, hepatitis C, syphilis, and HIV screening were negative. His renal function gradually declined over the following decade, resulting in ESRD. At 44 years of age, peritoneal dialysis was started for the treatment of ESRD, and an inguinal lymph node biopsy was performed to confirm the diagnosis, revealing the typical features of plasma cell-type MCD. Oral prednisolone (PSL) (10 mg/day) was initially administered; however, none of his clinical or laboratory parameters were fully resolved.

**Fig. 1:**
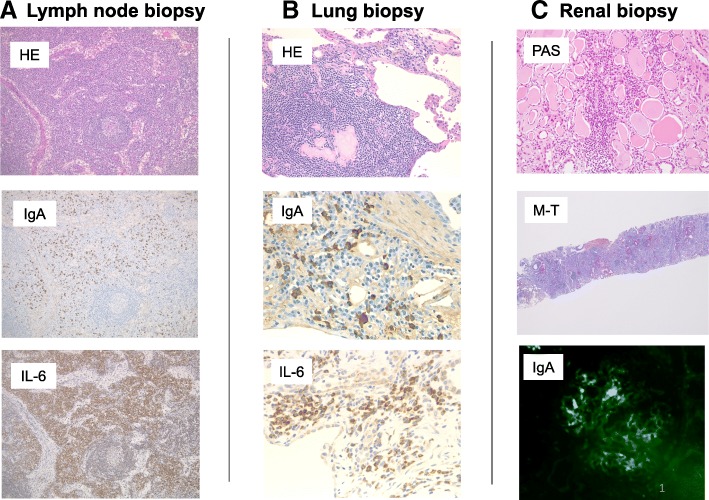
Histology and immunostaining. Specimens obtained from the inguinal lymph node, lung and kidney. **a** A microscopic examination of the inguinal lymph node revealed interfollicular expansion with plasmacytosis, compatible with plasma cell-type MCD. Numerous IgA- and IL-6-positive cells were detected. **b** A lung biopsy shows prominent infiltration of plasma cells with IgA- and IL-6-positivity. **c** Periodic acid-Schiff staining and Masson trichrome staining show ESRD; the stained blue area is the fibrous tissue in the ESRD kidney. Immunofluorescence staining of a renal biopsy specimen shows granular staining for IgA in the mesangium

At 45 years of age, he was referred to our hospital for further evaluation and treatment including living-donor KTx. To histopathologically determine the primary cause of ESRD (i.e., IgA nephropathy, AA amyloidosis, or other causes) and to make a pathological diagnosis of his interstitial lung disease, renal and lung biopsies, respectively, were performed. Similar to the immunostaining findings of the inguinal lymph nodes, which showed significant deposits of IgA and IL-6 (Fig. 1a), lung biopsy specimens showed plasma cell proliferation with positive staining for IgA and IL-6 (Fig. 1b), and renal biopsy specimens showed predominant IgA deposition in the glomerular mesangium (Fig. 1c). These findings led us to the possible diagnosis of MCD with lung involvement complicated by ESRD associated with IgA nephropathy. Since treatment with oral PSL failed to suppress the disease activity of MCD, the therapeutic regimen was changed to intravenous TCZ (8 mg/kg, every 2 weeks), which successfully controlled his MCD symptoms without the further exacerbation of his lung disease.

**Fig. 2:**
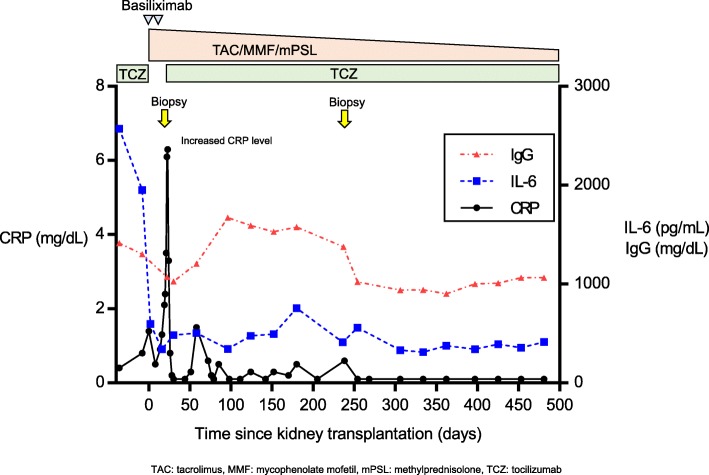
Clinical course after transplantation. Tapering of triple immunosuppression and sustained treatment with TCZ in an MCD transplant recipient. The IL-6 levels are currently maintained at 300–500 pg/ml, and the CRP and IgG levels are trending towards normal with no allograft rejection

At 50 years of age, he had recurrent peritonitis and therefore decided to undergo KTx. Preoperatively, tacrolimus (TAC) and mycophenolate mofetil (MMF) were administered starting 5 days before transplantation. TAC was administered at 0.1 mg/kg/day, and the trough level was maintained at 8–12 ng/mL for the first few weeks after transplantation. MMF was administered at a dosage of 1000 mg twice daily. In addition, induction with intravenous methylprednisolone (mPSL; 1 dose of 500 mg) and basiliximab (1 dose of 20 mg) therapy was administered on the day of transplantation. Basiliximab was also administered 4 days after transplantation. mPSL was gradually tapered to 40 mg by the end of the first post-transplantation week and switched to an oral formulation. To prevent over-immunosuppression, TCZ was discontinued after transplantation. However, the extension of the interval between TCZ administrations was associated with a slight increase in the patient’s CRP levels, but his constitutional symptoms did not reappear, and after the repeated administration of TCZ (8 mg/kg, every 2 weeks), improvements in his CRP levels were immediately noted (Fig. 2).

The patient’s IL-6 levels decreased dramatically after transplantation and are now maintained at 300–500 pg/ml under immunosuppression with a triple-drug regimen (TAC, MMF and mPSL) with TCZ (Fig. 2). In addition, the patient experienced increases in albumin, haemoglobin and haematocrit (data not shown), along with normalized CRP and IgG (Fig. 2).

On post-operative days 20 and 238, a transplant renal biopsy was performed and showed no rejection or virus infection. To evaluate for the presence of anti-HLA antibodies, a single-antigen bead assay (LABScreen Single Antigen) was performed. DSA was negative both before transplantation and on post-operative day 239.

At present, 17 months after KTx, the patient remains asymptomatic, and the allograft function has been preserved without evidence of rejection. In addition, haematologic follow-up has not demonstrated any significant adverse effects of TCZ (such as dyslipidaemia or myelosuppression) or cytomegalovirus (CMV) infection events.

## Discussion and conclusions

This case highlights two important clinical implications: MCD with ESRD can be successfully treated by KTx, and TCZ is useful for MCD and ensures a safe perioperative period during KTx.

In the past, the treatment of CD has included various therapeutic strategies, such as steroids, rituximab, and chemotherapy. However, since there is no standard therapy, treatment recommendations for CD can be difficult [3]. Recently, TCZ was shown to be effective for treatment and has been widely used in Japan since June 2005 [1, 2, 9, 10].

While renal involvement associated with MCD is rare, its manifestations can vary and include minimal disease changes, mesangial proliferative glomerulonephritis, AA amyloidosis, thrombotic microangiopathy (TMA) and IgA nephropathy [4, 5, 11–14]. In particular, IgA nephropathy associated with MCD is quite rare [11]. In our patient, in addition to the diagnosis of IgA nephropathy, numerous IgA-positive cells were detected in the lymph node and lung specimens. This result is consistent with those of a recent study, which found that the detection of increased immunohistochemical IgA expression can be useful for diagnosing CD [15]. Furthermore, ESRD as a complication of MCD has rarely been reported, and to our knowledge, only one report has described successful KTx for an MCD patient [6]. Murakami et al. reported an MCD patient with ESRD who underwent KTx. The patient developed acute rejection soon after KTx but was successfully treated with a steroid pulse, plasmapheresis, anti-CD3 and rituximab. The graft survival was 8 years, and the graft function remained excellent.

In patients with CD, increased IL-6 and CRP levels have been noted [1, 2, 12–14]. CD exacerbation is correlated with the levels of both IL-6 and CRP; IL-6 induces the synthesis of hepatic acute-phase reactants and increases CRP and IgG levels. In our patient, his IL-6 levels decreased dramatically after transplantation with induction therapy from 3480 pg/ml to 341 pg/ml. We suspect that induction therapy might have dramatically decreased his IL-6 levels.

IL-6 is a major cytokine involved in the progression of B cells to IgG-secreting plasmablasts and finally to plasma cells. In addition, IL-6 stimulates Th17 cells, which mediate inflammation and allograft rejection. Recent studies have suggested that TCZ can inhibit antibody production and reduce inflammation by targeting the IL-6/IL-6R pathway [16–18]. Jordan et al. reported on the efficacy of TCZ in reducing anti-HLA antibodies and improving the graft survival in highly HLA-sensitized patients who were resistant to other desensitization strategies. Furthermore, they offered TCZ treatment to chronic antibody-mediated rejection patients in whom other treatment options had failed, and significant reductions in DSA and stabilization of renal function were seen at 2 years [7, 8].

Another study suggested that TCZ may be useful for preventing post-transplant graft-versus-host disease (GVHD) [19]. Kennedy et al. reported that the addition of TCZ to the standard GVHD prophylaxis for patients who had received allogenic stem-cell transplantation was safe and associated with a very low incidence of GVHD.

The long-term use of TCZ is optimal for the treatment of MCD; thus, treatment with TCZ may allow KTx patients to reduce or discontinue their conventional immunosuppression and mitigate related side effects, such as hypertension, dyslipidaemia, de novo diabetes, infection, and malignancies [20]. We therefore expect immunosuppression after KTx to also be effective for MCD.

In summary, we suggested a treatment protocol for renal replacement therapy using TCZ for MCD patients with ESRD. While the IL-6R monoclonal antibody TCZ was shown to be effective for treating MCD, it may also be used to reduce the risk of chronic antibody-mediated rejection and thereby possibly improve long-term graft survival [7, 8]. Although the indications for elective KTx in MCD patients remain limited, continued success and reports of good outcomes may help establish KTx as an acceptable therapeutic option.

## Abbreviations

CD: Castleman disease
CNI: Calcineurin inhibitor
DSA: Donor-specific antibody
ESRD: End-stage renal disease
GVHD: Graft-versus-host disease
IL-6: Interleukin 6
IL-6R: Interleukin-6 receptor
KTx: Kidney transplantation
MCD: Multicentric Castleman disease
MMF: Mycophenolate mofetil
mPSL: Methylprednisolone
PSL: Prednisolone
TAC: Tacrolimus
TCZ: Tocilizumab
